# Assessment of the Impact of a Head-mounted Augmented Reality Low Vision Aid on Vision and Quality of Life in Children and Young People with Visual Impairment

**DOI:** 10.22599/bioj.345

**Published:** 2024-01-22

**Authors:** Emily Cottingham, Finnguala Burgum, Simon Gosling, Laura Woods, Anamika Tandon

**Affiliations:** 1 Sheffield Children’s Hosptial, UK

**Keywords:** Low vision, electronic low vision aid, quality of life

## Abstract

**Introduction:**

Electronic head-mounted low vision aids (LVAs) can help children and young people (CYP) to access schoolwork and leisure activities which they would otherwise struggle to be able to do with traditional optical or hand held LVAs. SightPlus uses a smartphone mounted in a virtual reality headset controlled using a Bluetooth joystick. It offers users 0.7–24.3× magnification alongside enhanced modes to maximise vision.

**Methods:**

Eighteen participants aged 8–16 years with reduced vision were given SightPlus to use at home for four weeks. Visual acuity was assessed with and without SightPlus along with reading performance, contrast sensitivity, functional vision and quality of life questionnaires.

**Results:**

Clinically significant improvements in distance vision (0.633logMAR SD ± 0.359), near vision (0.411logMAR SD ± 0.368), reading acuity (0.454LlogMAR SD ± 0.406) and critical print size (0.285logMAR ± 0.360) were seen when testing with SightPlus.

However, there was a mean decrease in contrast sensitivity and reading speed when using SightPlus. Despite this, nine out of the 14 patients included for analysis indicated a preference to continue to use SightPlus. Of note, younger participants were more likely to show a preference for using SightPlus. All seven CYP aged 10 or under wanted to continue to use SightPlus; in contrast, only two of the seven participants aged 11 or over wanted to continue.

**Conclusions:**

Like the results in adult populations, SightPlus has been found to improve CYP visual functions. Older participants were less likely to want to continue to use SightPlus, potentially suggesting they have found other methods for managing sight loss.

## Introduction

There are 24,000 children and young people (CYP) in the UK with moderate or severe visual impairment (VI) (RNIB, 2021). Low vision aids help CYP to maximise their vision.

Optical vision aids are commonly used but are often hand-held and tend to be limited to a fixed magnification. In addition, as magnification increases the field of view decreases. Consequently, research on the use of electronic low visual aids (e-LVA’s) for those with visual impairment is becoming increasingly imperative as health care services attempt to offer more effective alternatives.

In a study by Golubova et al. (2021) participants with low vision identified portability, variable magnification and reliability to be of highest importance when it came to designing their ideal low vision aid (LVA). e-LVA’s offer some of these benefits; they can accommodate near, intermediate and distance viewing alongside adjustable magnification. Head mounted options are hands-free enabling patients to access more activities.

Geruschat et al. (1999) assessed the use of three different head-mounted displays (HMD’s) in ten students (ages 12–21) and found that visual acuity and contrast sensitivity was significantly improved with the HMD’s. However, it was noted that HMD’s were cosmetically noticeable but that some students liked their appearance.

Cullham et al. (2004) compared four, head mounted e-LVA’s with participants usual optical aids and found variable results. Whilst some devices performed significantly better than traditional optical aids for distance viewing, one device led to a reduction in vision when compared to optical e-LVA’s. When looking at reading speed for N20 and N10 print, reading speed was reduced using the e-LVA’s in comparison to the optical LVA’s. The HMD’s were found to have an improved resolution for distance and intermediate tasks but no single device was superior across the participants highlighting the difficulties in designing and producing these devices.

In contrast, Wittich et al. (2018) found significant improvements in distance visual acuity, near reading acuity, critical print size and contrast sensitivity when using the head-mounted e-LVA, eSight in 51 participants. Wittich et al. (2018) also assessed functional vision with the eSight and found a significant improvement in the ability of participants to identify facial expressions with the eSight along with an improvement in overall visual ability and when measured with the Melbourne Low Vision Activities of Daily Living Index and Veterans Affairs Low Vision Visual Functioning Questionnaire.

However, head-mounted e-LVA’s are not without fault as highlighted by Ehrlich et al. (2017) who noted that the field of view being positioned close to the eyes can induce aesthenopic symptoms. In addition, images on the display may be of low resolution and can cause fatigue. There is also concern that HMD’s can be heavy, limit head motion and cause the slow tracking of objects (Erlich et al. 2017).

More recently smartphones have been used in e-LVA’s. Yeo et al. (2022) identified an improvement in distance, intermediate and near visual acuity alongside reading acuity, facial recognition and quality of life. However, there was a reduction in reading speed. Despite this 32 out of the 34 participants were interested in purchasing the device.

Crossland et al. (2019) investigated the effect of SightPlus, a head-mounted, augmented reality, smartphone-based e-LVA in adults and demonstrated improvements in visual acuity and contrast sensitivity. Although these improvements were found in the clinical application of SightPlus (during a 10–15-minute assessment), the study did not investigate the practical use in everyday life and tasks.

We were approached by the technological creators of SightPlus (Give Vision) to ask to run a similar study with children and young people with visual impairment. The aim of this study is to assess the effect of using the SightPlus on vision and quality of life in CYP with moderate and severe VI. In addition, it will assess the usability and acceptability of SightPlus in CYP.

## Methods

### Ethics

Ethical approval was obtained from the Health Research Authority (IRAS project ID: 291155) and authorised by the directorate of research and innovation at Sheffield Children’s NHS Foundation Trust.

The research was conducted in accordance with ICH Principles of good practice; the World Medical Association Declaration of Helsinki 1996 and the UK Policy Framework of Health and Social Care Research.

Informed consent was given by participants aged 16 years and by parent/guardians of those under 16 years. Assent was given by participants aged under 16 years.

### Participants

CYP aged 8-16 years who are cared for the in the eye department at Sheffield Children’s Hospital (SCH) were invited to take part. It is not recommended that children under eight years use SightPlus due to usability and the size of the headset. Participants had a moderate or severe visual impairment with a best corrected visual acuity of between 0.500logMAR and 1.300logMAR (as defined by World Health Organisation, 2019) when tested with both eyes open using LogMAR Crowded Keeler.

Participants were selected from a database of current patients referred from SCH to Sheffield visual impairment services. The hospital notes of all age-appropriate patients (n = 73) on the database were screened by Orthoptist (LW); those who fitted the inclusion criteria (n = 28) were allocated a three-digit pseudonymisation number (001 – 028). These were then used by a random number generator to select the order in which patients were invited to the study by Orthoptists SG or FB. Of the 28 patients approached by the study, 18 agreed to participate.

During the screening process patients were excluded if their visual acuity (VA) level at their most recent appointment (measured in the best seeing eye or both eyes open) fell outside of the defined parameters (0.500 – 1.300 logMAR); if they were unable to complete the clinical tests required by the study or where the investigators felt they would struggle to use SightPlus due to physical or cognitive difficulties; and if their parent/guardian lacked fluent written and spoken English. CYP with an implanted medical device such as a pacemaker or with a history of neurological conditions such as epilepsy or seizures were also excluded as per the manufacturer’s guidance.

### Materials

**Figure 1:**
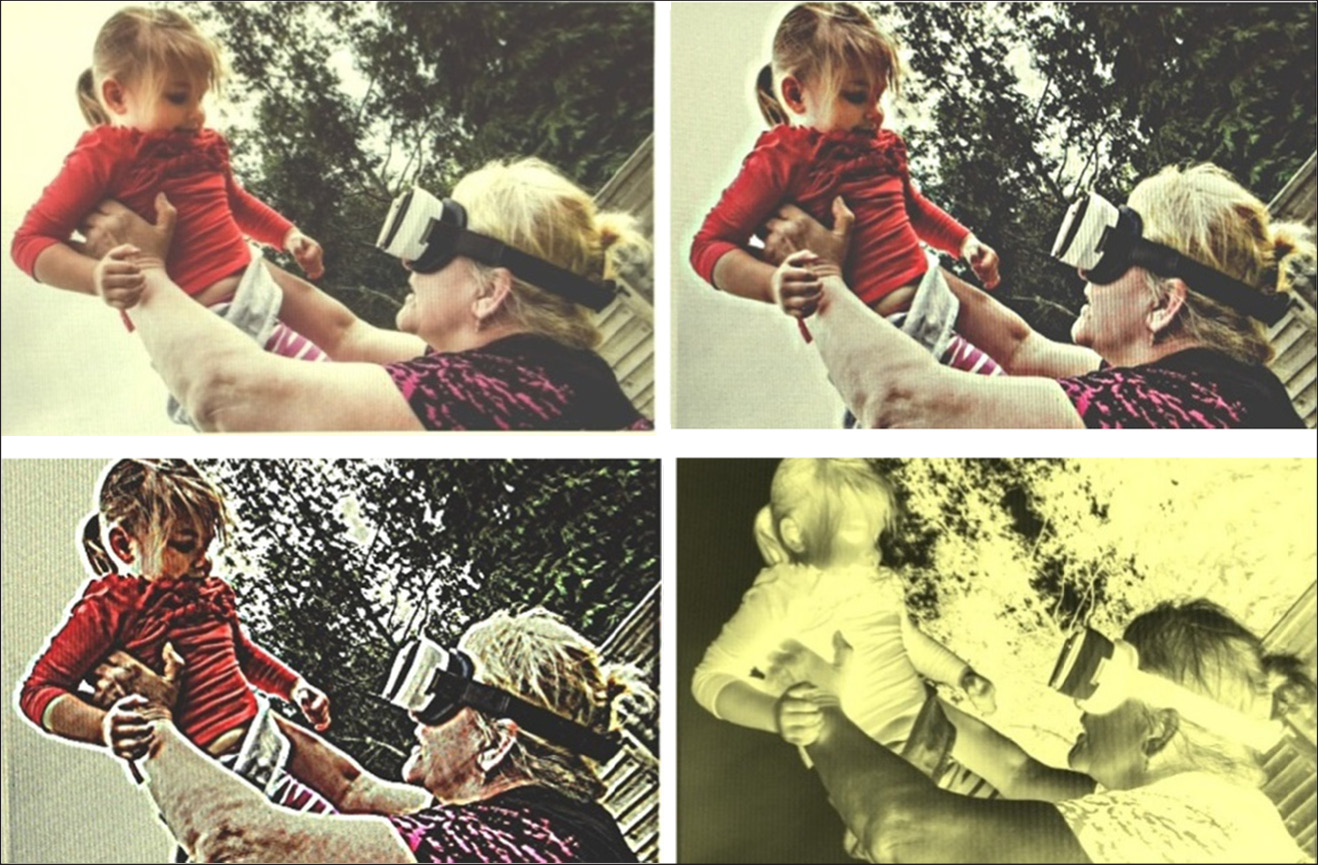
Different modes available in SightPlus. Top left: normal mode, top right: enhanced mode, bottom left: contrast mode, bottom right: inverted mode. Image reproduced with permission from Give Vision.

SightPlus uses a Samsung Galaxy 8 phone mounted in a Homido Prime virtual reality headset which is controlled using a handheld Bluetooth remote control/joystick. SightPlus weighs 465 g and has a screen resolution of 2960×1440. This provides users with a 110 degree (80 degree horizontal) field of view and approximately 0.7× 24.3× magnification. In addition, there are 5 modes that participants can use to enhance their vision. These are: normal, enhanced (provides sharper edges and smoother colours), contrast, inverted mode (inverted greyscale image) and text mode (yellow on black), examples of these modes can be seen in Figure 1.

### Procedure

**Figure 2:**
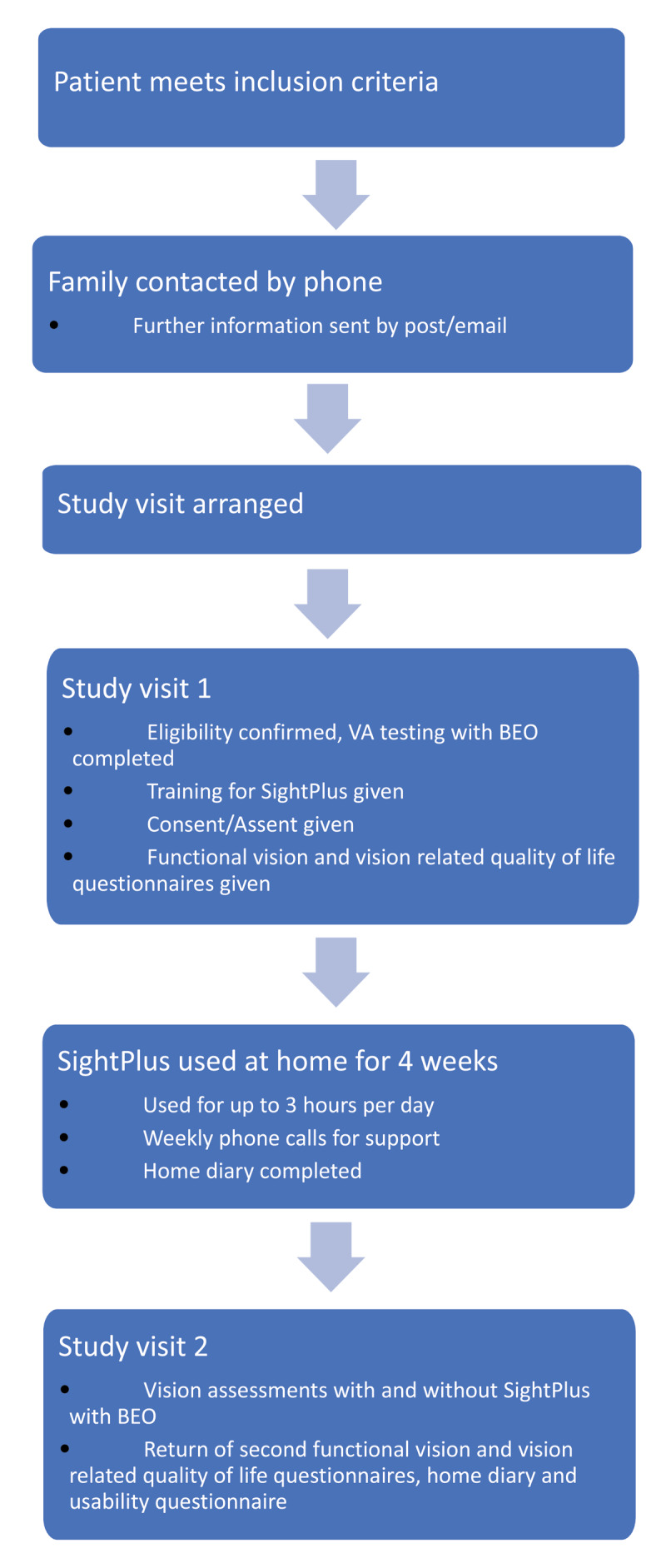
Flow chart showing participants involvement during the study.

The study took place over two appointments at Sheffield Children’s Hospital (SCH) eye department, four weeks apart. Figure 2 shows the participants involvement during the study, at visit one best corrected VA was assessed BEO to ensure the inclusion criteria was met. CYP were then trained to use SightPlus and were given it to take home and use up to three hours per day for four weeks. During visit two, visual assessments were undertaken (refractive correction in place) with and without SightPlus (order of testing was counterbalanced) and participants were free to choose the mode used without direction.

Distance VA was assessed using LogMAR crowded Keeler, letter-by-letter scoring was used with testing ending when a participant was not able to identify three letters on a line. If a participant was not able to identify the largest letters at 3 m the test was brought forward until it was possible to identify the letters. Near VA was assessed at 40 cm using the Sloan reduced logMAR.

Contrast sensitivity was assessed using the Evans Low Contrast Sensitivity Test at 1 m, the triplet was scored correctly when two out of the three letters were correctly identified.

The MNRead at 40 cm was used to assess the reading acuity size, which is the smallest print that can be read. In addition, the MNRead was used to assess the critical print size, this is the smallest print that can be fluently read and reading speed which is the speed at which the critical print size is read (recorded in words per minute).

Assessment of recognition of facial expressions was conducted using the NimStim Dataset, 12 images were presented at 50cm and participants were asked to identify the facial expression, whilst the number of errors the patient made was recorded. The NimStim Dataset provides a valid and reliable set of contemporary multiracial facial expressions (Tottenham et al. 2009). Due to the young age of the participants in the study the expressions included were limited to fear, sadness, anger and happiness. Equal numbers of each expression, a range of ethnicities and male and female faces were included.

Participants were given a Functional Vision Questionnaire (FVQ) and Vision-Related Quality of life Questionnaire (VQoL) appropriate to their age. These questionnaires provide a valid and reliable tool to allow the assessment of functional vision, social impact and for assessing the effectiveness of low vision rehabilitation (Rahi et al. 2011; Tadić et al. 2013 and 2016). Two copies of the questionnaires were given to the participants at visit one. Due to the Covid-19 pandemic restrictions participants completed the questionnaires at home to minimise time in the hospital environment. Questionnaires could be returned by post or at visit two. Participants were instructed to complete the first questionnaires as soon as possible and the second just before visit two. In order to encourage the return of the questionnaires, participants were offered a £15 voucher after the questionnaires were returned to thank them for their time. Participants were also offered travel expenses of up for £15 per family per visit.

### Analysis

Descriptive statistics were used to analyse the results.

## Results

Eighteen participants (three females and 15 males) took part in the study. The cohort consisted of children who had sight deteriorating conditions and those with congenital visual impairments. Four participants (numbers 002, 005, 026 and 027) were excluded from analysis as they chose to withdraw from the study prior to data completion. Participant 015 was excluded from analysis as their distance visual acuity varied by a clinically significant amount between visit one and visit two, however, their data relating to usage and the qualitive patient feedback was included.

Therefore, the analysis for change in visual performance, comparing with and without SightPlus at visit two was conducted for 13 participants and for qualitive results 14 participants were included.

The clinical diagnoses can be seen in Table 1.

**Table 1:** Details of VA comparing with and without use of the SightPlus and participants diagnosis. Table is arranged by participant age. Positive numbers indicate an improvement in acuity. Please note patient 015* was excluded from some analysis.


PARTICIPANT NUMBER	AGE (YEARS)	GENDER	CHANGE IN DISTANCE VA (LOGMAR)	CHANGE IN NEAR VA (LOGMAR)	PRIMARY DIAGNOSIS	NYASTAGMUS (Y/N)	WOULD CHOOSE TO KEEP SIGHTPLUS (Y/N)

023	8	M	0.525	0.400	Congenital Idiopathic Nystagmus	Y	Y

028	8	M	0.700	0.620	Subependymal grey matter heterotropia	Y	Y

019	9	M	0.650	0.380	Oculocutaneous Albinism	Y	Y

009	10	M	0.000	–0.200	Cerebral Palsy	Y	Y

012	10	M	0.550	0.660	Maternal substance misuse	Y	Y

008	10	F	1.300	1.140	Leber’s Amaurosis	Y	Y

013	10	F	0.525	0.240	Microphthalmia	Y	Y

016	11	M	0.500	0.400	Ocular Albinism	Y	N

007	11	M	0.950	0.160	Achromatopsia	Y	Y

015*	11	M	0.902	1.100	Oculocutaneous Albinism	Y	N

001	12	M	0.800	0.900	Stargardt’s	N	N

003	14	M	0.375	0.300	Stargardt’s	N	N

025	14	M	1.152	0.520	Bilateral optic chiasm glioma	N	Y

017	16	M	0.200	–0.120	Optic chiasm glioma	Y	N


### Clinical measures

**Figure 3:**
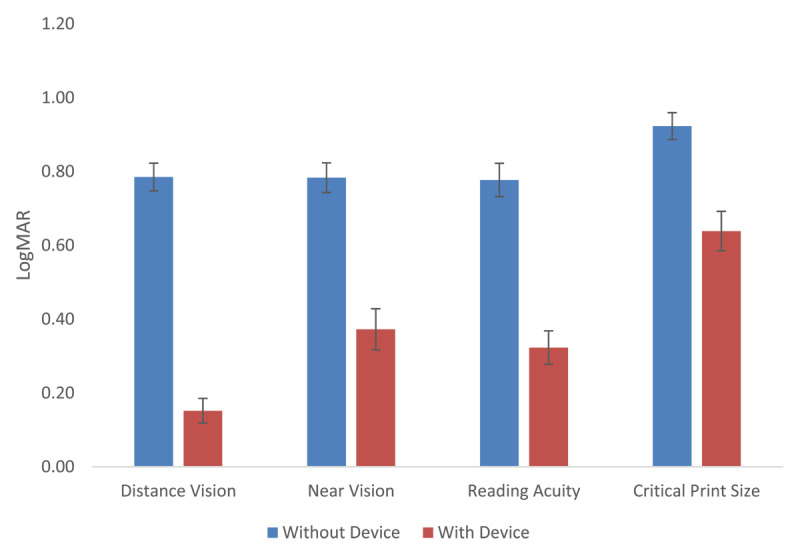
The comparison of distance visual Acuity, near visual acuity, reading acuity and critical print size with and without SightPlus.

Assessment of both near and distance VA (Figure 3) demonstrated a clinically significant improvement when tested using SightPlus in comparison to assessment without SightPlus. Twelve out of 13 participants had an improvement in distance VA when using SightPlus, the mean improvement in distance vision was found to be 0.633logMAR (SD ± 0.359).

Eleven participants had an improvement in near VA, with the mean improvement being 0.411logMAR (SD ± 0.368). A similar improvement in reading acuity was found (mean 0.454logMAR SD ± 0.406). As can be seen in Figure 3, the mean improvement in critical print size was smaller (0.285logMAR SD ± 0.360).

Only four participants demonstrated an improvement in contrast sensitivity. During the assessment CYP tended not to swap between different modes and generally only used the zoom function. The mean change in contrast sensitivity with the SightPlus was an overall reduction in contrast of 0.169 (SD ± 0.541).

### Functional measures

The average reading speed without the SightPlus was 61.8 words per minute (WPM) (SD ± 53.9), with SightPlus there was minimal reduction with the average reading speed 60.4 WPM (SD ± 43.2). Only six participants had an improvement in maximum reading speed when using SightPlus.

The ability of CYP to identify facial expressions improved when using the SightPlus. Out of a potential 12 errors, on average 2.4 (SD ± 2.3) errors were made without SightPlus, this improved to only 1.3 (SD ± 1.5) errors when using SightPlus.

### Quality of life

**Figure 4:**
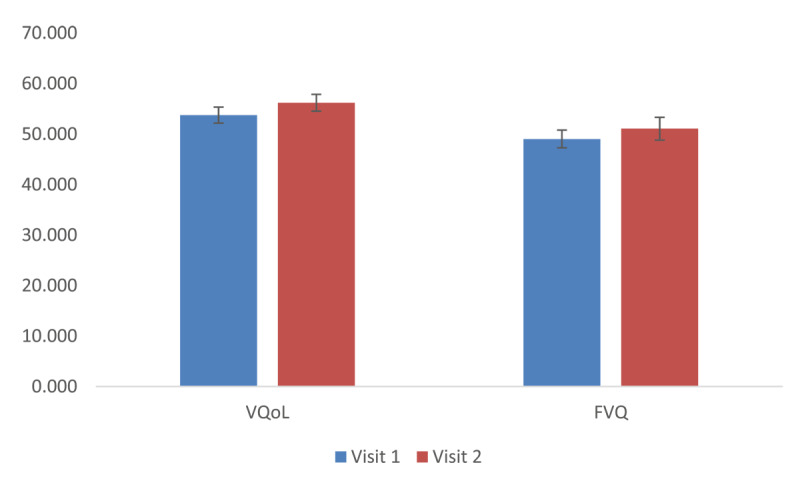
Comparison of vison related quality of life (VQoL)and function vision questionnaires (FVQ) at visit 1 and 2.

In view of the Covid-19 pandemic, time spent in the hospital had to be minimised, consequently the VQoL and FVQ were completed at home rather than in the clinic. This led to limited data being available as some participants did not return the questionnaires. Therefore, there are only nine participants who returned the VQoL and FVQ. As can be seen in figure 4 there is only a small improvement on both questionnaires completed at visit 1 and visit 2.

### Willingness to continue to use

Nine out of 14 participants indicated that they would like to have continued using SightPlus. All seven participants aged 10 or under wanted to continue using Sightplus, in contrast only two of the seven patients aged 11 or over wanted to continue with SightPlus.

**Figure 5:**
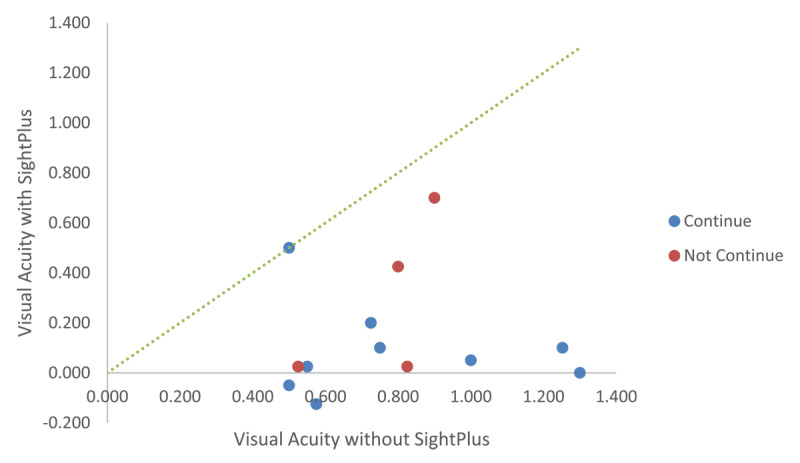
Comparison of distance visual acuity with and without SightPlus. Data points below the line indicate an improvement in VA when wearing the SightPlus, those on the line indicate no change.

Figure 5 shows that most participants had an improvement in distance VA with SightPlus but highlights that 2 out of the 4 participants who did not want to continue with SightPlus had a smaller improvement in visual acuity in comparison to those who wanted to continue.

As can be seen in Table 2 there was no clinically significant difference between the groups for near vision, critical print size of contrast sensitivity.

**Table 2:** Comparison of clinical measures for participants who did and did not want to continue using SightPlus.


	WANTED TO KEEP SIGHTPLUS		DID NOT WANT TO KEEP SIGHTPLUS	
	
	MEAN CHANGE	STANDARD DEVIATION	MEAN CHANGE	STANDARD DEVIATION

Distance vision	0.706	0.388	0.469	0.253

Near vision	0.429	0.369	0.370	0.419

Reading print size	0.489	0.386	0.375	0.499

Critical print size	0.278	0.342	0.300	0.455

Contrast sensitivity	0.161	0.616	0.188	0.394


**Figure 6:**
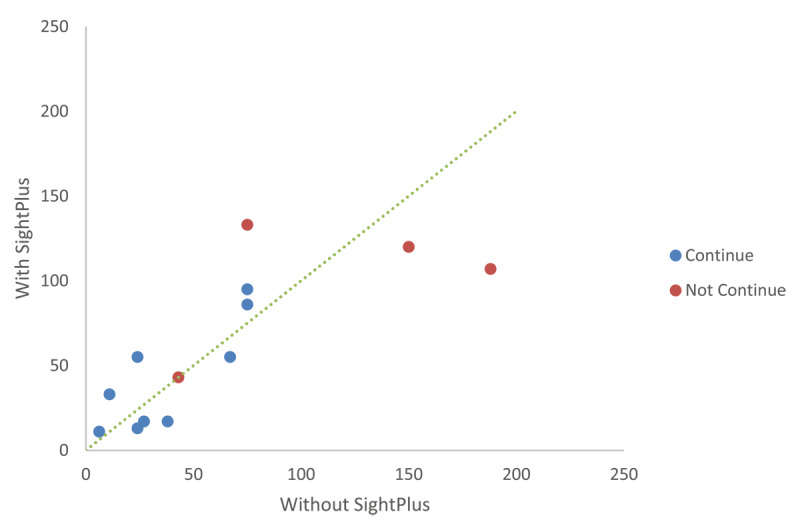
Comparison of reading speeds with and without SightPlus.

There was variability in reading speed with SightPlus, some participants found using it improved their reading speed, whilst others had a reduction compared to their reading speed with refractive correction alone (figure 6). Of the CYP who did not want to continue with SightPlus, generally, higher reading speeds were seen. However, this could be due to age or indicate that these participants had adapted to their visual impairment and thus didn’t see as much benefit to SightPlus.

### Useability

**Figure 7:**
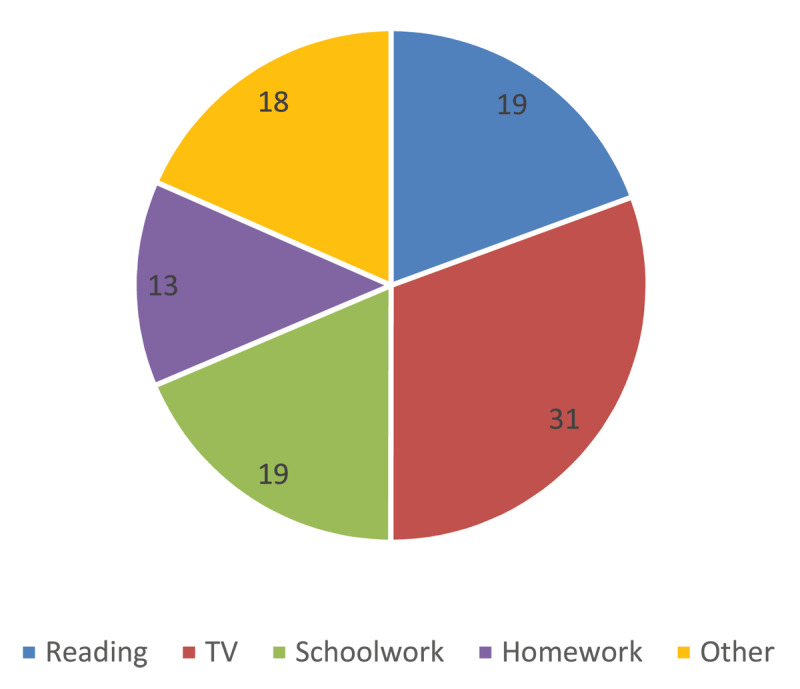
Percentage of time spent completing different activities when using SightPlus.

Participants used SightPlus for a range of activities, figure 7 shows what percentage of the time CYP used SightPlus for different activities. Watching TV was the most popular activity to take part in when using the SightPlus. On the clinical measures distance vision was the area where the greatest improvement was seen.

**Figure 8:**
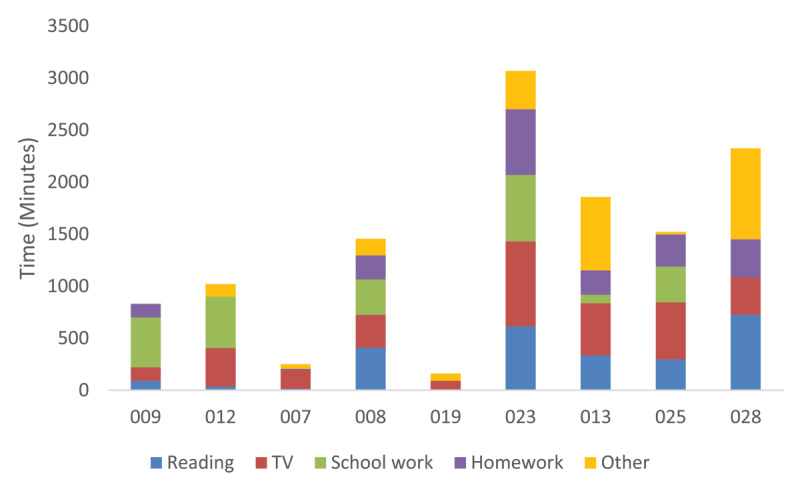
Length of time participants who wanted to continue using SightPlus used it for during the study.

**Figure 9:**
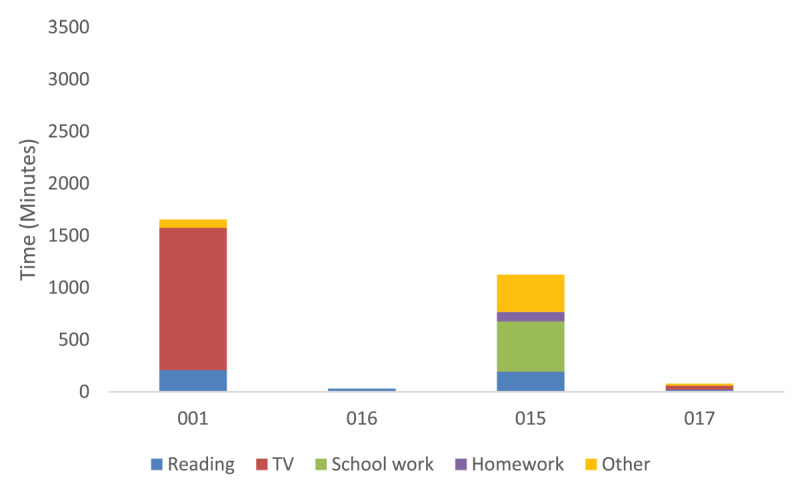
Length of time participants who did not want to continue using SightPlus used it for during the study.

Figures 8 and 9 show how long each participant used SightPlus for during the four-week trial period. There is a wide range between the participants in relation to the number of minutes that SightPlus was used for. However, some CYP who did not want to keep it used it very little which may suggest that they were quick to judge if SightPlus was right for them. One of the two participants who used SightPlus more frequently but did not want to continue after the study had ended was found to have a clinically significant improvement in their vision whilst using SightPlus. The other participant only had an improvement in vision of 0.200logMAR.

### SightPlus feedback

Qualitative feedback was collated from home diaries, telephone calls and parent/participant feedback forms.

During the weekly telephone calls participants were asked to report side effects. In total half of the participants (seven) reported side effects with the most common being motion sickness, headaches and dizziness which were all reported by two different participants. In addition, one participant reported that SightPlus was uncomfortable. Despite this, the participant still wanted to continue to use SightPlus after the study.

Nine participants commented that the best part of using SightPlus was the zoom, three enjoyed trying the different modes and two found it fun.

There was a greater range of issues with reported when participants were asked to identify the worst thing about SightPlus as can be seen in Table 3.

**Table 3:** Issues reported by CYP when using SightPlus.


ISSUE	NUMBER OF PARTICIPANTS REPORTING

Heavy/big	5

Not working/set up takes too long	4

Cumbersome	3

Camera quality/blur	3

Battery life	3

Dizzy	1


Some families had extremely positive feedback about SightPlus:

‘*[patient] has loved the device. We have discussed how this could improve his school experience. If he had this device forever, he wouldn’t always have to sit at the front of class. Also, in assembly he always can hear what’s going on but can’t see. We also set up a deck chair on garden and for the first time he could zoom in to see bus numbers. Another massive difference we noticed was when playing Mario karts or watching TV he could sit on the settee across the room rather than on the floor right in front of the settee.*’

But others were disappointed and found SightPlus more of a hinderance:

‘*Didn’t live up to expectations. It is very heavy – the quality of the images is poor, text doesn’t stay still on the page which makes reading harder.*’

One patient (025) commented on their feedback form that they would not want to wear the device in the street. They were not explicit about whether this was related to motion or the cosmesis of the device when it is being worn.

## Discussion

### Clinical measures

As expected, using SightPlus lead to an improvement in VA. The mean improvement of distance visual acuity in this study was 0.633logMAR (SD ± 0.359). The adult population assessed by Crossland et al. (2019) found a similar mean improvement in vision when using SightPlus of 0.63logMAR (SD ± 0.34). Given this similar finding it can be concluded that children are able to use SightPlus as efficiently as the adult population.

Other studies looking at HMD’s in predominantly adult populations found greater improvements, which suggests that other HMD’s may offer greater visual benefits. When testing with the eSight Eyewear Wittich et al. (2017) found the improvement in vision was 0.74logMAR (SD ± 0.285). A larger improvement in distance vision was found when testing vision with a smartphone-based LVA in a study by Yeo et al. (2022) where the mean improvement was 0.98logMAR.

The mean improvement in near VA in this study was 0.411logMAR (SD ± 0.368). In an adult population Crossland et al. (2019) improvement was less at only 0.10logMAR. Other eLVAs have given greater improvement in near vision, using a wearable smartphone-based LVA Yeo et al. (2022) found a mean improvement of 0.90logMAR. Wittich et al. (2018) found an improvement of 0.66logMAR when testing patients with the eSight.

In the current study overall contrast sensitivity was reduced when using the SightPlus – this was an unexpected finding. In contrast in an adult population Crossland et al. (2019) found that the SightPlus lead to an improvement of 0.38 log units, however, only 58% of participants in this study used the “normal” mode, with the remaining using other modes to further enhance their vision. The current study allowed the participants to choose which mode on the device they utilised but unfortunately did not record which mode was used during the assessment. However, from participant feedback the normal mode with zoom was the preferred function for the CYP. This allows the authors to speculate that the way in which the CYP prefer to use SightPlus has led to a mean reduction in contrast sensitivity which would affect the visual experience of the CYP.

### Functional measures

There was variability regarding reading speed, including several CYP who had a reduction in reading speed. This is to be expected given that Crossland et al. (2019) also found a reduction in reading speed of 17.11 words per minute with using SightPlus. When testing with the wearable smartphone-based LVA Yeo et al. (2022) found only a small mean improvement of 4.25 words per minute. The benefit SightPlus offers CYP is the clinically significant improvement in reading acuity which will allow them access to documents that would otherwise need to be augmented or be inaccessible.

Wittich et al. (2018) are the only other authors who assessed face perception; they found an improvement in the ability of participants to identify the gender and emotional expression in images when using eSight. Although using different methodology, the current study also found an improvement with fewer mistakes identifying expressions occurring when using SightPlus.

### Quality of life

A small improvement in quality of life and functional vision has been reported by CYP after using SightPlus in the current study. Unfortunately, there is limited data available as participants were required to complete the questionnaires at home due to Covid-19 restrictions; consequently, full data was only available for nine participants.

Wittich et al. (2018) found a significant improvement in activities of daily living and low visual functioning when assessed using the Melbourne Low Vision Activities of Daily Living Index and Veterans Affairs Low Visual Functioning Questionnaires respectively. In contrast, when assessing Low Vision Quality of Life Yeo et al. (2022) did not find an overall significant difference with and without the wearable smartphone-based LVA. However, there was a significant improvement in participants aged under 40.

The smaller improvement in the current study could indicate that CYP are more adapted to alternative techniques and thus adapting to newer technology did not improve their quality of life.

### Willingness to continue

Overall 64% of participants were willing to continue to use SightPlus, with a trend for participants aged 10 or under being more willing to continue to use. This may be that older children were more self-conscious using SightPlus, or that they saw less benefit having had more time or resources to have developed strategies for managing sight loss.

In comparison to adults, more CYP were keen to use SightPlus, with only 47% of adults interested in using SightPlus (Crossland et al. 2019). However, adults were not given the opportunity to take SightPlus home to use for everyday activities as part of the study. When testing with a different wearable smartphone-based LVA Yeo et al. (2022) found 94.12% willing to continue to use them.

### Usability

There is limited published data regarding use of HMD e-LVA’s in real world settings. Students taking part in the current research enjoyed exploring the visual scene in the classroom and identifying other students facial expressions and watching things out of the window. It is understandable that this would create such fascination given that HMD’s including SightPlus provide opportunities for CYP to experience things that they may otherwise never have seen. In the written feedback from parents, it is clear to see how much CYP enjoyed the new experiences that SightPlus can offer.

### SightPlus feedback

Some CYP wanted to continue to use SightPlus. This is not surprising given its benefits for distance viewing which is an area that has been identified where current LVAs are lacking (Golubova et al. 2021). However, none of the participants purchased the device at the end of the study. There is no clear data about why this was the case, it may have been the expense of a costly device; the cosmesis or comfort of the device; or the restrictions of the technology potentially inducing motion sickness/ headaches and reducing clarity. This would be an interesting area to explore more in further research using HMD’s. The weight and blur noticed by CYP have also been suggested as potential problems by Ehrich et al. (2017). Another problem identified by this cohort of CYP was the set up being slow. In an adult population 63% of participants would prefer a LVA with quick access for more spontaneous tasks (Golubova et al. 2021). Further work is needed to improve SightPlus so it better meets the needs of CYP.

## Conclusion

SightPlus offers CYP with low vision the opportunity to enhance their vision and for some visual functions such as distance vision they are able to use it as efficiently as an adult population. For other functions such as contrast sensitivity CYP do not see the improvements that can be seen in adult populations using e-LVA’s.

CYP have found SightPlus has given them the opportunity to engage with their surroundings in ways which would otherwise be impossible for them. However, SightPlus is not without fault and CYP were quick to point out difficulties with its size, speed, clarity and battery life. It is of note that primary school aged children were more accepting of SightPlus and more willing to want to use it in the longer term.

In conclusion, SightPlus offers an additional option for CYP with low vision to help them access school and leisure activities but further work should be done to increase acceptability and usability.
